# A Modified Rife Algorithm for Off-Grid DOA Estimation Based on Sparse Representations

**DOI:** 10.3390/s151129721

**Published:** 2015-11-24

**Authors:** Tao Chen, Huanxin Wu, Limin Guo, Lutao Liu

**Affiliations:** College of Information and Communication Engineering, Harbin Engineering University, Harbin 150001, China; E-Mails: chentao@hrbeu.edu.cn (T.C.); wuhuanxin1990@163.com (H.W.); liulutao@hrbeu.edu.cn (L.L.)

**Keywords:** direction of arrival (DOA) estimation, sparse representations, eigenvalue decomposition (EVD), off-grid, Rife algorithm

## Abstract

In this paper we address the problem of off-grid direction of arrival (DOA) estimation based on sparse representations in the situation of multiple measurement vectors (MMV). A novel sparse DOA estimation method which changes MMV problem to SMV is proposed. This method uses sparse representations based on weighted eigenvectors (SRBWEV) to deal with the MMV problem. MMV problem can be changed to single measurement vector (SMV) problem by using the linear combination of eigenvectors of array covariance matrix in signal subspace as a new SMV for sparse solution calculation. So the complexity of this proposed algorithm is smaller than other DOA estimation algorithms of MMV. Meanwhile, it can overcome the limitation of the conventional sparsity-based DOA estimation approaches that the unknown directions belong to a predefined discrete angular grid, so it can further improve the DOA estimation accuracy. The modified Rife algorithm for DOA estimation (MRife-DOA) is simulated based on SRBWEV algorithm. In this proposed algorithm, the largest and sub-largest inner products between the measurement vector or its residual and the atoms in the dictionary are utilized to further modify DOA estimation according to the principle of Rife algorithm and the basic idea of coarse-to-fine estimation. Finally, simulation experiments show that the proposed algorithm is effective and can reduce the DOA estimation error caused by grid effect with lower complexity.

## 1. Introduction

Approaches for direction of arrival (DOA) estimation have been widely studied [1,2,3,4,5,6]. In recent years, sparse representations and reconstruction theory have also been applied to DOA estimation [7,8,9,10,11,12,13]. The ability of multi-source resolution and efficient estimation in a few snapshots are two important advantages of DOA estimation using sparse theory. Theoretically, only one snapshot is needed to estimate parameters in sparse representations theory, but in practical applications more snapshots are sampled in order to improve the accuracy of DOA estimation. This problem is the so-called multiple measurement vectors (MMV) problem [14]. In [7,8], the Bayesian compressive sensing (BCS) framework is used in the DOA estimation problem. In paper [15], a joint recovery algorithm to estimate the angle spectrum was proposed, but the computational burden of this method becomes larger with the increasing number of snapshots and the estimation results may be influenced more easily by signal-to-noise ratio (SNR). In order to reduce the computational burden and improve estimation precision, there are some classical methods to solve the MMV problem, such as L1-SVD [16], R-GBCD+ [17], M-FOCUSS and L1-ACCV [18]. These algorithms all take datum correlations of different snapshots into consideration. The L1-ACCV algorithm can transform the MMV problem into a single measurement vector (SMV) model through an array cross-correlation vector (ACCV) [18]. Inspired by the L1-ACCV and L1-SVD algorithms, we propose a sparse representations based on weighted eigenvectors (SRBWEV) algorithm which can change the MMV problem into a SMV problem by using the linear combination of eigenvectors of array covariance matrix in signal subspace as a new SMV.

However, no matter what kind of sparsity-based methods are used, they all face a common off-grid estimation problem that true DOAs may not be on the discretized sampling grid [19] in some situations. Although the model is sparse in a continuous angular domain, we must construct a finite dictionary by sampling this domain with a predefined sampling grid in order to apply the sparse theory framework. Therefore, the true DOAs of targets are almost surely not located exactly on a subset of these grid points. This phenomenon leads to a model mismatch that results in a degradation of the performance. Of course, for higher DOA estimation accuracy a smaller grid spacing is required, which leads to a higher computational cost. If we reduce the number of grid points, the off-grid probability of the spatial source increases and the resultant DOA estimation accuracy decreases [20]. To compromise between the DOA estimation accuracy and the computational cost, some algorithms such as multiresolution grid refinement [16], coarse-to-fine DOA estimation [20], SRTLS, SBI algorithms and SOMP-LS algorithm [21] were proposed to deal with these problems, but these algorithms still have a large computational burden. The basic idea of these algorithms is that the approximate sparse solution is found in the first step and then grid or optimization search algorithm refinement is adopted. Inspired by the frequency estimation approach based on FFT using the Rife algorithm and M-Rife algorithm [22], we introduce in this paper a simple approach that is the modified Rife algorithm for DOA estimation based on the proposed SRBWEV algorithm (MRife-DOA). Firstly we finish an approximate on-grid DOA estimation by using the SRBWEV algorithm. Then we further modify the DOA estimation results by using a modified Rife algorithm which uses neighboring atoms on both sides of estimated on-grid atoms and two new additional atoms produced according to estimate on-grid atoms, so the estimation accuracy can be improved effectively.

This paper is concerned with high accuracy DOA estimation with low complexity based on sparse representations. The remainder of this paper is organized as follows: we present DOA estimation based on the SRBWEV algorithm in Section 2. In Section 3, refining of the DOA estimation using the modified Rife algorithm is proposed. The simulation results and some discussions are given in Section 4. Finally, we conclude our work in Section 5.

## 2. Signal Model of DOA Estimation Based on Sparse Representations 

### 2.1. Input Signal Model

Suppose K far-field narrowband signals impinging on a uniform linear array (ULA) which has M antenna array elements spacing d from directions $\theta =[\theta_{1}\;\theta_{2}\;\cdots \;\theta_{K}]$. The received signal can be expressed as:

(1) y(l)=A(θ)s(l)+n(l),         l=1,2,⋯,L

where $A(\theta )=[a(\theta_{1})\;a(\theta_{2})\;\cdots \;a(\theta_{K})]\in {\mathbb{C}}^{M\times K}$ is the array manifold matrix, $a(\theta_{k})={\left[1,e^{-j\frac{2\pi d}{\lambda }\sin\theta_{k}},\cdots ,e^{-j\frac{2\pi (M-1)d}{\lambda }\sin\theta_{k}}\right]}^{T}$ is the steering vector of the array, ${\left[\cdot \right]}^{\text{T}}$ denotes the transpose, λ is the carrier wavelength, s(l) is incident signals, n(l) is additive complex Gaussian noise with zero mean, spatially and temporally uncorrelated with $E[n(l)n^{H}(m)]=\delta_{l.m}\sigma^{2}I$, ${\left(\cdot \right)}^{\text{H}}$ stands for Hermitian transformation, L is the number of snapshots.

### 2.2. Sparse Representations

The signal sparse representations mean a signal can be represented in an ultra-complete redundancy dictionary. Then we find the best linear combination of atoms to represent the original signal. An ultra-complete redundancy dictionary to represent original signal y(l) of Equation (1) may be established as follows:

(2) $$\bar{A}=[a(\alpha_{1})\;a(\alpha_{2})\;\cdots \;a(\alpha_{P})]$$

where $\bar{A}$ is a known ultra-complete redundancy dictionary matrix with M<<P. This dictionary is an angles set that denotes a sampling grid of all possible DOAs. According to Equation (2) we can rewrite the signal model of Equation (1) to group L snapshots as:

(3) $$Y=\bar{A}X+N$$

where Y=[y(1), y(2),⋯ ,y(L)] is a matrix of size M×L, X=[x(1), x(2),⋯ ,x(L)] is a matrix of size P×L and N is a noise matrix of size M×L. Then we find K nonzero coefficients in the vector x(l) and zero coefficients in the remaining P−K, *i.e.*, the sparsity in the angle space denotes that only a few atoms from the dictionary can be required to match the measurements. Here K<P condition is satisfied.

### 2.3. The Proposed SRBWEV Algorithm

To calculate the sparse solution by using the new algorithm, we utilize an important property of the relation between eigenvectors of an array covariance matrix and the steering vectors in the signal model (1) firstly. The property is described as “Based on signal model of Equation (1) the eigenvectors of array covariance matrix $R_{Y}$ in signal subspace is equal to linear combination of steering vectors $a(\theta_{k})\;$ (k=1,2,⋯,K)” (for the proof, see [23]). From the property we know that we can use eigenvector or eigenvectors’ linear combination in signal subspace as a new measurement vector instead of the matrix Y in order to solve MMV problem. Meanwhile we learn that the property can be satisfied in the situation of both correlated and uncorrelated incident signals. So we generalize the scheme of SRBWEV algorithm.

Input: The received signal matrix Y, and a sparse representations dictionary matrix $\bar{A}=\{a(\alpha_{p}),p=1,2,\cdots ,P\}\in {\mathbb{C}}^{M\times P}$.

(1) EVD for array covariance matrix $R_{Y}$;
(2) Determine the number of the larger eigenvalues (N) and eigenvectors corresponding to N larger eigenvalues such as $e_{1}$,$e_{2}$,⋯,$e_{n}$,⋯,$e_{N}$;
(3) Make a single measurement vector e combined by linear combination of eigenvectors $e_{1}$,$e_{2}$,⋯,$e_{n}$,⋯,$e_{N}$,
  (4) $$e=\sum_{n=1}^{N}\frac{{\text{ζ}}_{n}}{\text{ζ}}e_{n}$$
  where ${\text{ζ}}_{n}$(n=1,2,⋯,N) is the N larger eigenvalues of array covariance matrix $R_{Y}$ and $\text{ζ}={\text{ζ}}_{1}+\cdots +{\text{ζ}}_{n}+\cdots +{\text{ζ}}_{N}$;
(4) Search the index atoms in atomic dictionary by using OMP algorithm;
(5) Output: DOA.

Here, we make a weighted linear combination using eigenvalues for eigenvectors of the covariance matrix. Meanwhile, we use the OMP algorithm [20] to solve the sparse solution, so the SRBWEV algorithm can change the MMV problem into a SMV problem so as to reduce sparse solution calculation iterations.

## 3. Refining the DOA Estimation Using the Rife Algorithm

A limitation of the above model is that it assumes that the unknown directions fall on the predefined angular grids which the precision of DOA estimation is dependent on, but designing the fine grid for the dictionary can increase the computational burden. In order to further improve the precision of DOA estimation, we propose a simple algorithm based on the Rife algorithm which is based on correlation of two neighboring distinct dictionary atoms.

### 3.1. Correlation of Two Distinct Dictionary Atoms in Atomic Dictionary

We define correlation coefficient μ corresponding to ratio of the absolute value of inner product between two distinct dictionary elements (e.g., $a(\alpha_{p})$ and $a(\alpha_{q})$) and the product of each Euclidean distance of these two distinct dictionary elements [24]:

(5) $$\mu (\alpha_{p},\alpha_{q})=\frac{\left|\langle a(\alpha_{p}),a(\alpha_{q})\rangle \right|}{\left\|a(\alpha_{p})\right\|\left\|a(\alpha_{q})\right\|}$$

where $a(\alpha_{p})$ and $a(\alpha_{q})$ are dictionary elements. Fixing the $\alpha_{p}$ value which is the relative initial angle and defining $\Delta \alpha =\alpha_{q}-\alpha_{p}$, then we have:

(6) $$\begin{array}{l} \mu (\alpha_{p},\alpha_{p}+\Delta \alpha )=\frac{\left|\langle a(\alpha_{p}),a(\alpha_{p}+\Delta \alpha )\rangle \right|}{\left\|a(\alpha_{p})\right\|\left\|a(\alpha_{p}+\Delta \alpha )\right\|}\\ \;=\frac{\left|\sum_{m=1}^{M}e^{j\frac{2\pi (m-1)d}{\lambda }(\sin\alpha_{p}-\sin(\alpha_{p}+\Delta \alpha ))}\right|}{M}\\ \;=\left|\frac{\sin(\pi dM\sin(\sin\alpha_{p}-\sin(\alpha_{p}+\Delta \alpha ))/\lambda )}{M\sin(\pi d(\sin\alpha_{p}-\sin(\alpha_{p}+\Delta \alpha ))/\lambda )}\right| \end{array}$$

Here, we only consider the main lobe width. Considering Δα≈0 and $\sin\alpha_{p}-\sin(\alpha_{p}+\Delta \alpha )\approx 0$, so Equation (6) can be changed as the following:

(7) $$\left|\frac{\sin(\pi dM\sin(\sin\alpha_{p}-\sin(\alpha_{p}+\Delta \alpha ))/\lambda )}{\pi dM(\sin\alpha_{p}-\sin(\alpha_{p}+\Delta \alpha ))/\lambda }\right|=\frac{1}{\sqrt{2}}$$

Then, we obtain:

(8) $$\left|\sin\alpha_{p}-\sin(\alpha_{p}+\Delta \alpha )\right|=\frac{1.39\lambda }{\pi dM}$$

(9) $$\left|\sin\alpha_{p}-\sin(\alpha_{p}+\Delta \alpha )\right|=\left|2\cos(\frac{\alpha_{p}+\alpha_{p}+\Delta \alpha }{2})\sin(\frac{\Delta \alpha }{2})\right|\approx \cos\alpha_{p}•\Delta \alpha_{0.5}$$

(10) $$\Delta \alpha_{mb}=2\Delta \alpha_{0.5}=\frac{50.7\lambda }{Md\cos\alpha_{p}}{(}^{\circ })$$

**Figure 1:**
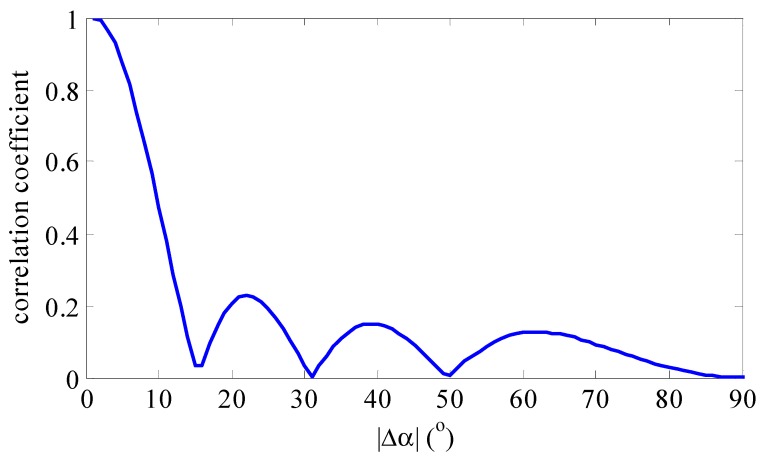
The correlation coefficient between two atoms with $\alpha_{p}=0{}^{\circ}$.

**Figure 2:**
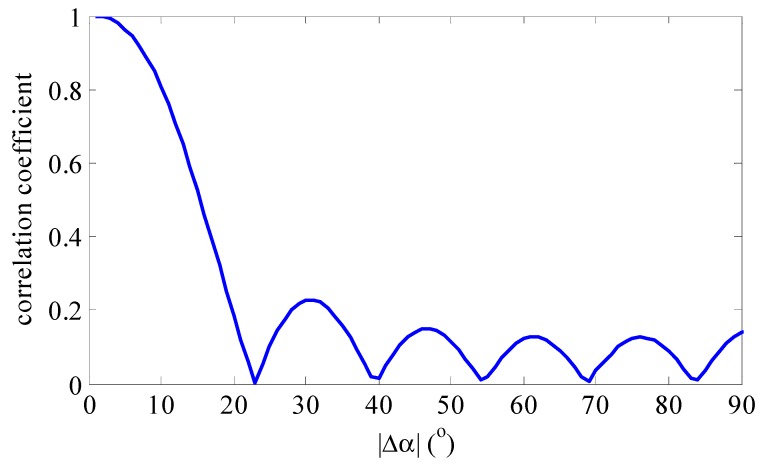
The correlation coefficient between two atoms with $\alpha_{p}=-60{}^{\circ}$.

Here, $\Delta \alpha_{mb}$ stands for the main spectral bandwidth and $\Delta \alpha_{0.5}$ stands for half of the main lobe width. From Equation (10), we learn that neighboring atoms in sparse dictionary have a strong correlation which is dependent on the number of array elements M, carrier frequency or wavelength λ, array spacing d and relative initial angle $\alpha_{p}$. The larger the relative initial angle is, the wider the main lobe is. For example, Figure 1 and Figure 2 show the correlation coefficient as a function of $\left|\alpha_{q}-\alpha_{p}\right|$ with the different relative initial angle $\alpha_{p}$. These simulation figures are obtained under the condition of using a ULA with eight antenna elements spaced at λ/2, so we can utilize the correlation to further improve the DOA estimation precision. Meanwhile, these figures show that the correlation coefficient between two neighboring atoms approaches the maximum value of 1 when the sampling resolution for the sampling grid increases.

**Figure 3:**
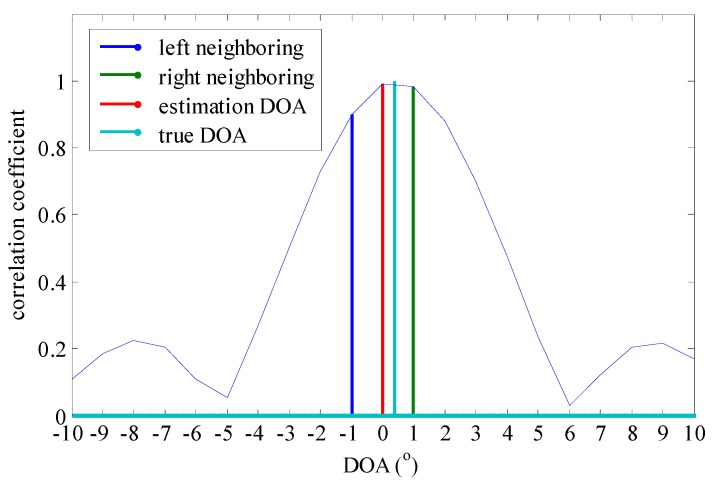
Off-grid and on-grid DOA estimation relationship.

In order to find the relationship between inner products, we further take an example of enlarging the correlation coefficient result around a nearby relative initial angle 0°. We assume there is a ULA with 20 antennas uniformly spaced with an antenna distance of λ/2. The grid is of 1° resolution with the grid points constituting the set {−90°,−89°,⋯,90°} and the true DOA of incident signal is set 0.4° which is an off-grid angle. The simulation results are shown in Figure 3 where estimation     DOA=0° (on-grid DOA estimation) is obtained by using the SRBWEV algorithm. Meanwhile, we give contour of the inner product from −10° to 10° and two neighboring inner products corresponding to −1° and 1° near the maximum inner product. From Figure 3, we know the neighboring inner products on both sides of the true DOA are larger than other inner products. The DOA estimation algorithm based on sparse representations chooses the nearest atom to match the true DOA. Inspired by this finding, we may choose three on-grid inner products which include the max and two neighboring values located on the left and right sides of the maximum to further estimate DOA by using the Rife algorithm.

### 3.2. The Principle for the Rife Algorithm

The Rife algorithm was initially proposed to complete frequency estimation based-DFT. The correlation between neighboring atoms is very similar to the relationship for neighboring frequency spectrum amplitude to a certain extent. We expect that the Rife algorithm can eliminate the grid effect by exploring the neighboring atoms’ relationship, so we will introduce Rife algorithm for calculating the DOA.

(11) $${\hat{\alpha }}_{c}=\alpha_{0}+\alpha_{res}\cdot \text{β}\cdot \frac{\left|B(\alpha_{0}+\alpha_{res}\cdot \text{β})\right|}{\left|B(\alpha_{0})\right|+\left|B(\alpha_{0}+\alpha_{res}\cdot \text{β})\right|}$$

Here, $\alpha_{res}$ denotes the grid resolution and $\alpha_{0}$ stands for the estimated on-grid DOA which are achieved by some algorithms based on sparse representations, such as L1-SVD, M-FOCUSS, L1-ACCV and the proposed SRBWEV algorithm. B(α) stands for the inner product operation between atom vectors corresponding to angle α in dictionary and the residual in the OMP algorithm.

However, there are some disadvantages in DOA estimation using the Rife algorithm directly. From the principle of the Rife algorithm and numerical simulation experiments, we learn the estimation performance is excellent when the incident signal’s DOA lies in the middle of two discrete on-grid angles. In this situation the error is smaller than with direct estimation using sparse representations, but when the true DOA of an incident signal is approximately or equal to the on-grid angle, the estimation error may be larger than with direct estimation using sparse representations. In the worst case the error may be reach half of the resolution for the grid.

Inspired by [22], we can modify the standard Rife algorithm for DOA estimation. Since we know if the true DOA lies in middle of the grid resolution, the estimation performance is good, so we can find two new atoms in order to make the true DOA lie in the middle of these two new atoms when the estimation DOA based on the standard Rife algorithm is approximated to the on-grid estimation angle, so we propose two loops for the modified Rife algorithm. The coarse estimation is realized to obtain the on-grid angle in the first loop and the fine estimation can be utilized to obtain an off-grid estimation. Here, we give the scheme for the modified Rife algorithm:

Input: The received signal matrix Y and a sparse representations dictionary matrix $\bar{A}=\{a(\alpha_{p}),p=1,2,\cdots ,P\}\in {\mathbb{C}}^{M\times P}$ with angle resolution $\alpha_{res}=\left|\alpha_{i+1}-\alpha_{i}\right|$.

(1) Use the SRBWEV algorithm we obtain the on-grid DOA estimation $\alpha_{i}$ and two neighboring on-grid DOAs $\alpha_{i+1}$ and $\alpha_{i-1}$;
(2) Use the Rife algorithm we obtain the off-grid coarse DOA estimation $\alpha_{EC}$;
(3) The Rife algorithm is modified:
  If $\left|\alpha_{EC}-\alpha_{i}\right|<\frac{1}{3}\alpha_{res}$, use the Rife algorithm secondly.
  (a) Compute two new inner products:
  (12) $${\text{ξ}}_{l}=\frac{\langle r,a(\alpha_{i-0.5})\rangle }{\left|r\right|•\left|a(\alpha_{i-0.5})\right|}$$
  (13) $${\text{ξ}}_{r}=\frac{\langle r,a(\alpha_{i+0.5})\rangle }{\left|r\right|•\left|a(\alpha_{i+0.5})\right|}$$
  (b) Obtain the off -grid fine DOA estimation:
  If ${\text{ξ}}_{l}\geq {\text{ξ}}_{r}$:
  (14) $$\alpha_{E}=\alpha_{i}-\frac{\alpha_{res}}{2}+\alpha_{res}\frac{{\text{ξ}}_{r}}{{\text{ξ}}_{l}+{\text{ξ}}_{r}}$$
  else:
  (15) $$\alpha_{E}=\alpha_{i}+\frac{\alpha_{res}}{2}-\alpha_{res}\frac{{\text{ξ}}_{l}}{{\text{ξ}}_{l}+{\text{ξ}}_{r}}$$
  else:
  (16) $$\alpha_{E}=\alpha_{EC}$$
(4) Output: DOA= $\alpha_{E}$.

Here, r denotes the residual in the OMP algorithm, $a(\alpha_{i-0.5})$ the vector corresponding to the angle $\alpha_{i}-\frac{1}{2}\alpha_{res}$ and $a(\alpha_{i+0.5})$ the vector corresponding to the angle $\alpha_{i}+\frac{1}{2}\alpha_{res}$ are two newly found atoms. Another problem is that the contour and properties of the main lobe are different from the DFT spectrum of. First of all, from Equation (10), we can know that the bandwidth of the main lobe is ascertained by the number of array elements M and relative initial angle $\alpha_{p}$, so we must guarantee that the $\alpha_{res}<\Delta \alpha_{mb}$ condition is satisfied. If not, the Rife algorithm is not effective. Generally speaking, DOA lies in [−60°,60°] in most of practical applications, so we can ignore the effect of $\alpha_{p}$. On the other hand, if $\alpha_{res}$ is too small, *i.e.*, the grid is so fine, that the difference for the normalized inner products for two neighboring on-grid atoms is very small. Here we can further modify the Rife algorithm. We can add a regularization factor ρ as a correction item. The Equations (11), (14) and (15) can be modified as follows:

(17) $${\hat{\alpha }}_{c}=\alpha_{0}+\alpha_{res}•\text{β}•\frac{\left|B(\alpha_{0}+\alpha_{res}•\text{β})\right|}{\left|\rho •B(\alpha_{0})\right|+\left|B(\alpha_{0}+\alpha_{res}•\text{β})\right|}$$

(18) $$\alpha_{E}=\alpha_{i}-\frac{\alpha_{res}}{2}+\alpha_{res}\frac{{\text{ξ}}_{r}}{\rho •{\text{ξ}}_{l}+{\text{ξ}}_{r}}$$

(19) $$\alpha_{E}=\alpha_{i}+\frac{\alpha_{res}}{2}-\alpha_{res}\frac{{\text{ξ}}_{l}}{{\text{ξ}}_{l}+\rho •{\text{ξ}}_{r}}$$

Regularization factor ρ can be determined by sampling grid spacing $\alpha_{res}$. If $\alpha_{res}$ is small, then ρ can be assigned a large value, and *vice versa*. According to numerical experiments, if $\alpha_{res}\approx \frac{\Delta \alpha_{mb}}{10}$, then ρ can be set in [1.5,5].

## 4. Simulation Experiments

In the following, we present some simulations to verify the theoretical results. We consider a ULA with eight antennas, uniformly spaced with an antenna distance of λ/2. The basic simulation parameters are set as follows. The number of snapshots is L=100. The regularization factor is set to be ρ = 3. The root mean square error (RMSE) is defined by Equation (20):

(20) $$\mathrm{RMSE}=(\frac{1}{J}\sum_{j=1}^{J}{\left({\hat{\theta }}_{j}-\theta_{k}\right)}^{2}{)}^{\frac{1}{2}}$$

where J is the times of independent Monte Carlo simulations, ${\hat{\theta }}_{j}$ stands for the DOA estimation value of the true DOA $\theta_{k}$ for the jth trial. One thousand independent Monte Carlo simulations are carried out for each SNR varied from −10 dB to 20 dB with 5 dB step. The true DOA is selected randomly in independent Monte Carlo simulations.

**Figure 4:**
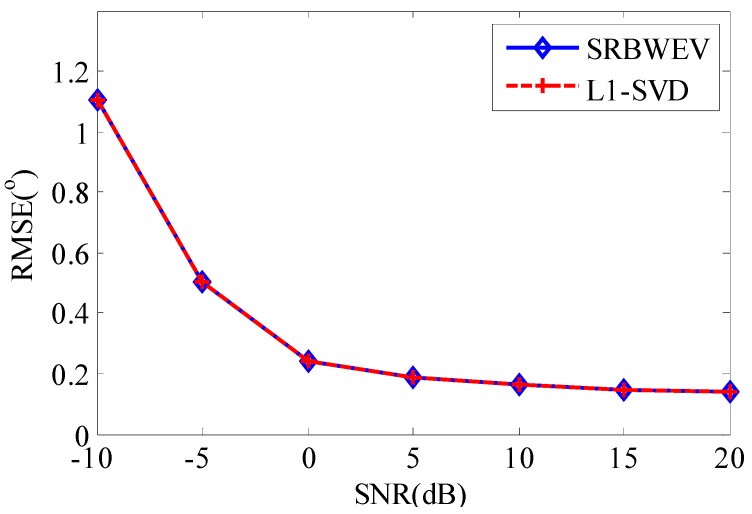
DOA estimation with grid spacing 0.5°.

**Figure 5:**
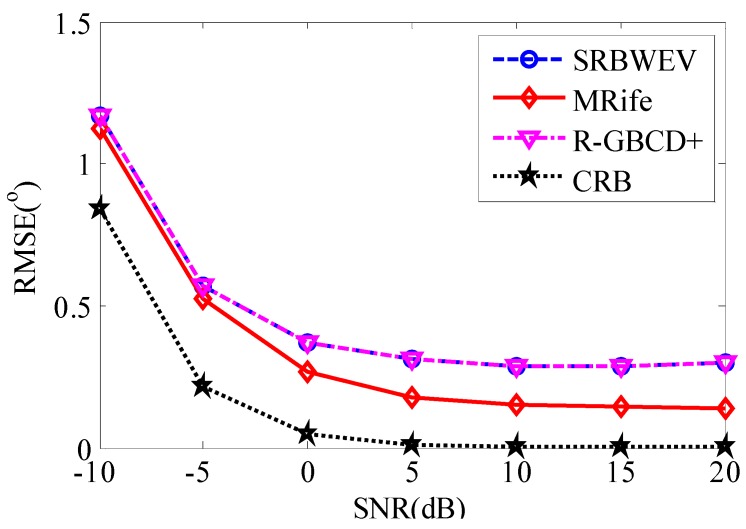
DOA estimation using modified Rife algorithm with grid spacing 1°.

In Figure 4 and Figure 5, SRBWEV stands for the new proposed algorithm called SRBWEV. L1-SVD stands for L1-SVD, R-GBCD+ stands for the R-GBCD+ algorithm, CRB stands for the Cramer-Rao bound (CRB) and MRife stands for the modified Rife algorithm for DOA estimation based on the SRBWEV algorithm.

In Figure 4, the grid spacing is equal to 0.5° compared with 1° in Figure 5. Figure 4 shows SRBWEV is effective for DOA estimation. It has almost the same performance as the L1-SVD algorithm in the case of 0.5° spacing with the same SNR. Compared with L1-SVD, the SRBWEV algorithm has a smaller computational burden.

In Figure 5, we obtain the estimation performance for MRife-DOA based on the SRBWEV algorithm. From the simulation results, we know the angle measurement error can be decreased by using the MRife algorithm. The accuracy of the MRife algorithm will be nearly improved by two-fold as compared to the *R*-GBCD+ algorithm and SRBWEV algorithm without the Rife modification. In other words, we have the same performance as Figure 4 in the case of grid spacing 1° instead of 0.5° grid spacing. Meanwhile, the computational cost is reduced by half.

**Figure 6:**
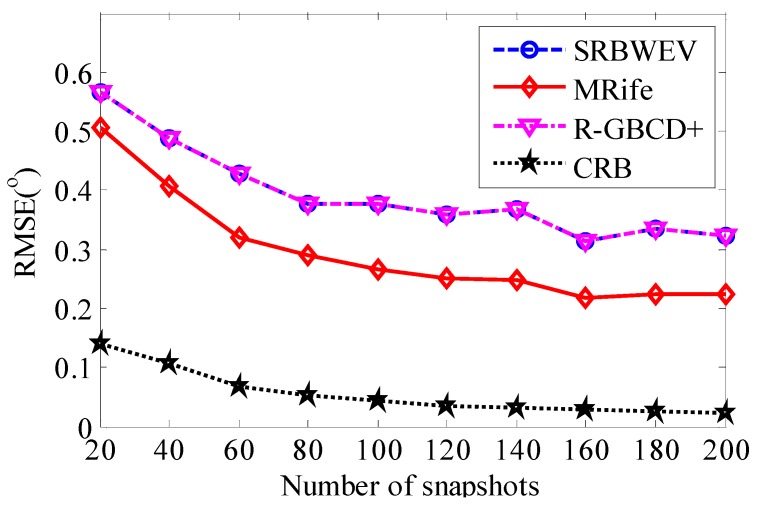
RMSE of DOA estimation *versus* number of snapshots.

SNR is set to 0 dB. One thousand independent Monte Carlo simulations are carried out for each number of snapshots varied from 20 to 200 in steps of 20. Figure 6 shows the RMSE of DOA estimation *versus* number of snapshots. It can be seen that the RMSE of the MRife algorithm is smaller than with the other two algorithms and the MRife algorithm can achieve better performance than the other two algorithms.

The grid is divided in the range of −60° to 60° in spacing of 1°. SNR is set to 0 dB. One hundred Monte Carlo simulations are carried out for each number of snapshots varied from 50 to 200 in steps of 50. Table 1 shows the average running time *versus* the number of snapshots. It can be seen from Table 1 that the running time of the SRBWEV algorithm is the shortest. The running time of the MRife algorithm is longer than that of the SRBWEV algorithm, but shorter than the other two R-GBCD+ and L1-SVD algorithms. Therefore, MRife algorithm can achieve better estimation performance with lower complexity.

**Table 1:** The running time *versus* the number of snapshots.

Number of Snapshots	MRife	SRBWEV	R-GBCD+	L1-SVD
50	0.0017 s	0.0015 s	0.0145 s	4.9925 s
100	0.0014 s	0.0012 s	0.015 s	4.9916 s
150	0.0016 s	0.0014 s	0.0136 s	4.9594 s
200	0.002 s	0.0018 s	0.0159 s	5.0757 s

## 5. Conclusions

In this paper, inspired by the Rife algorithm for frequency estimation, we propose a new DOA estimation method from coarse to fine estimation, named the modified Rife algorithm for DOA estimation (MRife-DOA) based on the SRBWEV algorithm which is a sparse decomposition based on weighted eigenvectors for DOA estimation. The proposed algorithm can decrease the computational burden for grid refinement in sparse representations for DOA estimation. Meanwhile, based on the principle that the eigenvectors of the covariance matrix in signal subspace are equal to a linear combination of steering vectors, we propose the SRBWEV approach which changes the MMV problem to a SMV one in order to reduce the number of sparse solution calculation iterations. The performance of the DOA estimation is close to that of the L1-SVD algorithm. Finally the simulation results of estimation accuracy and some discussions are shown. These analyses and trials illustrate the proposed algorithm is effective and may be applied to practical applications in the near future. However, mutual coupling has not been taken into account in this paper and this will be the subject of our future work.
